# Quinoa whole grain diet compromises the changes of gut microbiota and colonic colitis induced by dextran Sulfate sodium in C57BL/6 mice

**DOI:** 10.1038/s41598-018-33092-9

**Published:** 2018-10-08

**Authors:** Wei Liu, Yu Zhang, Bin Qiu, Shoujin Fan, Hanfeng Ding, Zhenhua Liu

**Affiliations:** 1 Shandong Center of Crop Germplasm Resources, Shandong Academy of Agricultural Sciences, Jinan, 250100 China; 2 Institute of Agro-Food Science and Technology, Shandong Academy of Agricultural Sciences, Jinan, 250100 China; 3 School of Public Health and Health Sciences, University of Massachusetts, Amherst, 01003 USA; 4 College of Life Science, Shandong Normal University, Jinan, 250014 China

## Abstract

A plethora of evidence highlights that the dysbiosis of gut microbiota is a critical factor for inflammatory bowel disease (IBD). Both *in vivo* and *in vitro* studies have demonstrated that quinoa possesses potential prebiotic effects. The present study aims to examine the potential in using quinoa to ameliorate the dysbiosis and colitis induced by dextran sodium sulfate (DSS). A total of 40 C57BL/6 mice were fed either an AIN-93M diet or a quinoa-based diet, separately. Colitis was induced for 10 animals/dietary group with a 5-days exposure to 2.5% DSS. The clinical symptoms were monitored every other day, and the gut microbiota was characterized by 16S rRNA gene sequencing. The results indicated that consumption of quinoa lessened clinical symptoms as indicated by the reduced disease activity index and the degree of histological damage (*P* < 0.05). As expected, the DSS treatment induced significant dysbiosis of gut microbiota in mice on an AIN-93M diet. However, compared to mice fed the AIN-93M diet, the consumption of quinoa alleviated the DSS-induced dysbiosis remarkably, as indicated by increased species richness and diversity, decreased abnormal expansion of phylum *Proteobacteria*, and decreased overgrowth of genera *Escherichia/Shigella* and *Peptoclostridium* (*P* < 0.05). The relative abundances of *Firmicutes* and *Bacteroidetes* were less altered in mice fed with quinoa comparing to those mice fed the AIN-93M diet. In summary, the consumption of quinoa suppressed the dysbiosis of gut microbiota and alleviated clinical symptoms induced by DSS, indicating the potential to utilize quinoa as a dietary approach to improve intestinal health.

## Introduction

Inflammatory bowel disease (IBD), categorized as either Crohn’s disease (CD) or ulcerative colitis (UC)^1,2^, has reached epidemic levels worldwide^3,4^. IBD is directly associated with an increased risk of colorectal cancer, but there are still no effective means to treat it. In fact, long-term used of traditional immunosuppressive agents for IBD treatment, e.g. azathioprine, might lead to higher incidence of various cancers^5^. Thereby, it is of critical importance to develop strategies to prevent the occurrence of IBD^6,7^.

Although no study has convincingly determined any specific bacterial group or strain that definitely causes IBD, it is well accepted that the dysbiosis of gut microbiota is a critical contributing factor in the etiopathogenesis of IBD^8^. IBD patients exhibit a significant reduction of biodiversity and stability of gut microbiota^4^. They also have a reduced abundance of phyla *Firmicutes* and *Bacteriodetes* accompanied with a considerably increased abundance of *Proteobacteria* and *Actinobacteria*^9,10^. Some specific bacterial groups, such as *Enterobacteriaceae* and *Desulfovibrio*, were found to be expanded in IBD patients^11,12^. Studies using animal models provided more direct evidence: after inoculation with microbiota from mice with colitis, the healthy recipient can develop intestinal inflammation^13^.

Diet is a direct mediator for gut microbiota and thereby offers an attractive means for the prevention of IBD. *Chenopodium quinoa* Willd (quinoa), an edible grain-like crop from the Andes region of South America, is rich in high quality protein, vitamins and minerals, and in particular possesses a wide range of various polysaccharide^14^. *In vitro* studies have suggested that quinoa has a prebiotic effect, including promoting the growth of beneficial bacteria and the production of SCFAs (Short chain fatty acids)^15^. Polysaccharides from quinoa have demonstrated immune-regulating activity in animal studies^16^. All of the evidences suggests that quinoa may have beneficial effects on intestinal health.

In this study, utilizing the dextran sodium sulfate (DSS)-induced colitis animal model, we investigated to what extent the consumption of quinoa alleviated the dysbiosis of intestinal microbiota and reduced the symptoms of DSS-induced colitis. This was done with a consideration of developing dietary strategies for the prevention of IBD.

## Results

### Body physiology and pathological indices

**Figure 1:**
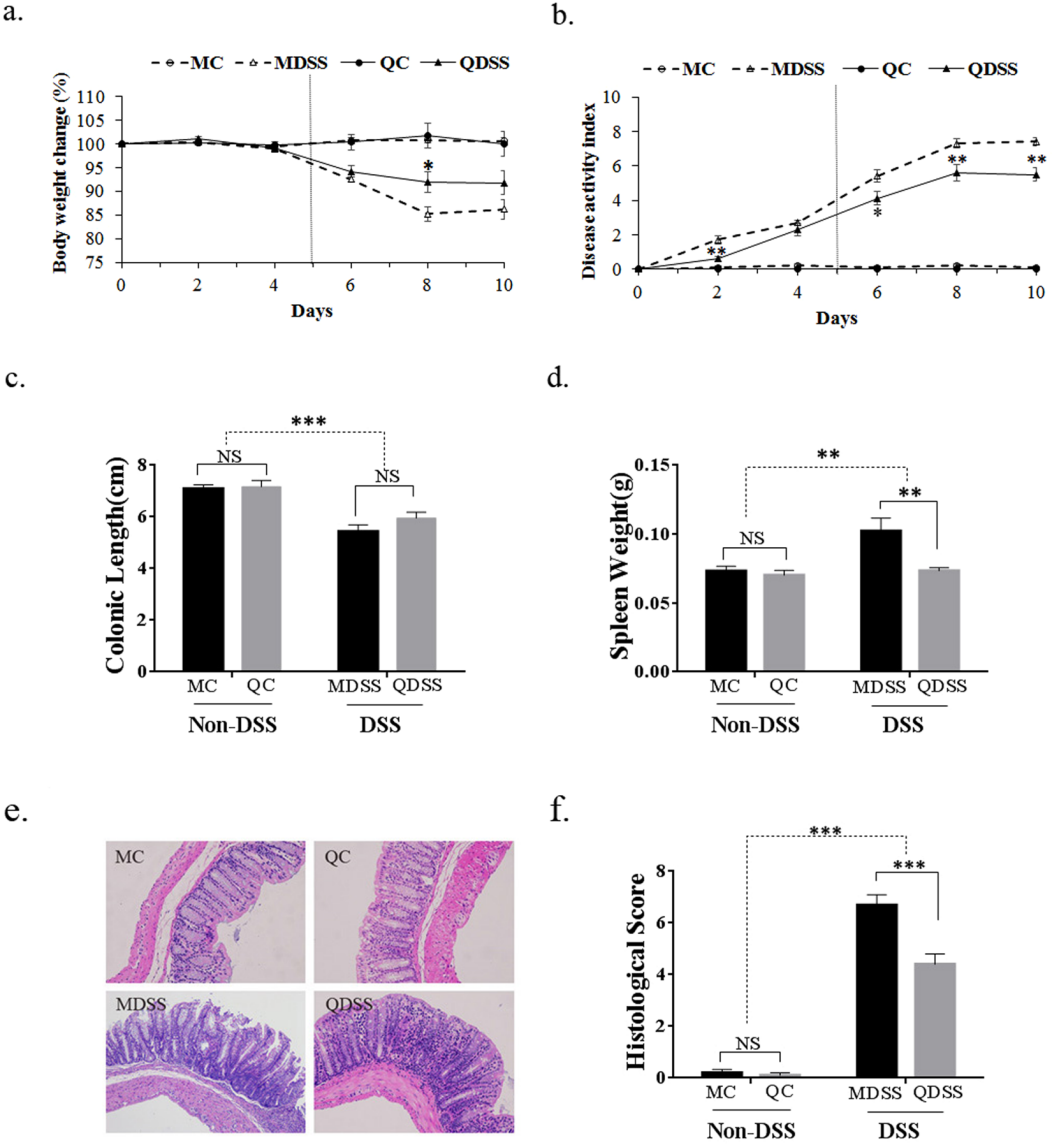
Body physiology and pathological indices: (**a**) relative body weight changes and (**b**) disease activity index (DAI) scores monitored every two days over the 10 days (MDSS and QDSS mice was treated with 5 days DSS exposure in drinking water and followed by another 5 days tap water); (**c**) colon lengths, (**d**) spleen weights, (**e**) histological analysis and (**f**) histological severity scores of the different treatment mice. Comparisons of various anatomical measurements among four groups were made by two-way analysis of variance (ANOVA), followed by Fisher’s LSD post hoc tests. ^*^Indicates significant difference with *P* < 0.05, ^**^Indicates *P* < 0.01 and ^***^Indicates *P* < 0.001; NS indicates lack of statistically significant difference. In (**a**) and (**b**) the statistically differences were only marked within the DSS-treated or Non-DSS control groups (MC *vs*. QC; MDSS *vs*. QDSS).

In this study, groups of mice exposed to DSS developed clinical symptoms, including body weight loss, diarrhea, and rectal bleeding. As shown in Fig. 1a,b, the body weights and DAI scores of non-DSS controls remained stable through the whole 10 days, while the body weights of DSS-treated groups decreased significantly from day 4 and the DAI increased significantly from day 2. When comparing the two DSS-induced groups (MDSS vs. QDSS), it was determined that the body weight loss of QDSS mice was less than that of MDSS mice from day 6 to day 10, and the difference was statistically significant on day 8. The QDSS mice also showed lower DAI scores than MDSS mice from day 2 to the end of the study, with statistical significances shown on days 2, 6, 8 and 10 (*P* < 0.05).

As shown in Fig. 1c,d, the DSS-treated groups displayed reduced colon lengths (Fig. 1c, *P* < 0.001) and enlarged spleens (Fig. 1d, *P* = 0.0076). In comparing the two DSS-treated groups, the spleen weight of mice on the AIN-93M standard diet (MDSS) was significantly higher than that of mice on the quinoa diet (QDSS) (*P* = 0.0288). No significant difference in colonic length was observed between MDSS and QDSS groups. Histological evaluation showed that DSS treatment significantly increased colonic inflammation (Fig. 1e), whereas the consumption of quinoa attenuated the tissue damages, as indicated by a lower histological score for the QDSS group compared to the MDSS group (Fig. 1f, *P* < 0.001).

### Cecal microbial diversity indices

**Figure 2:**
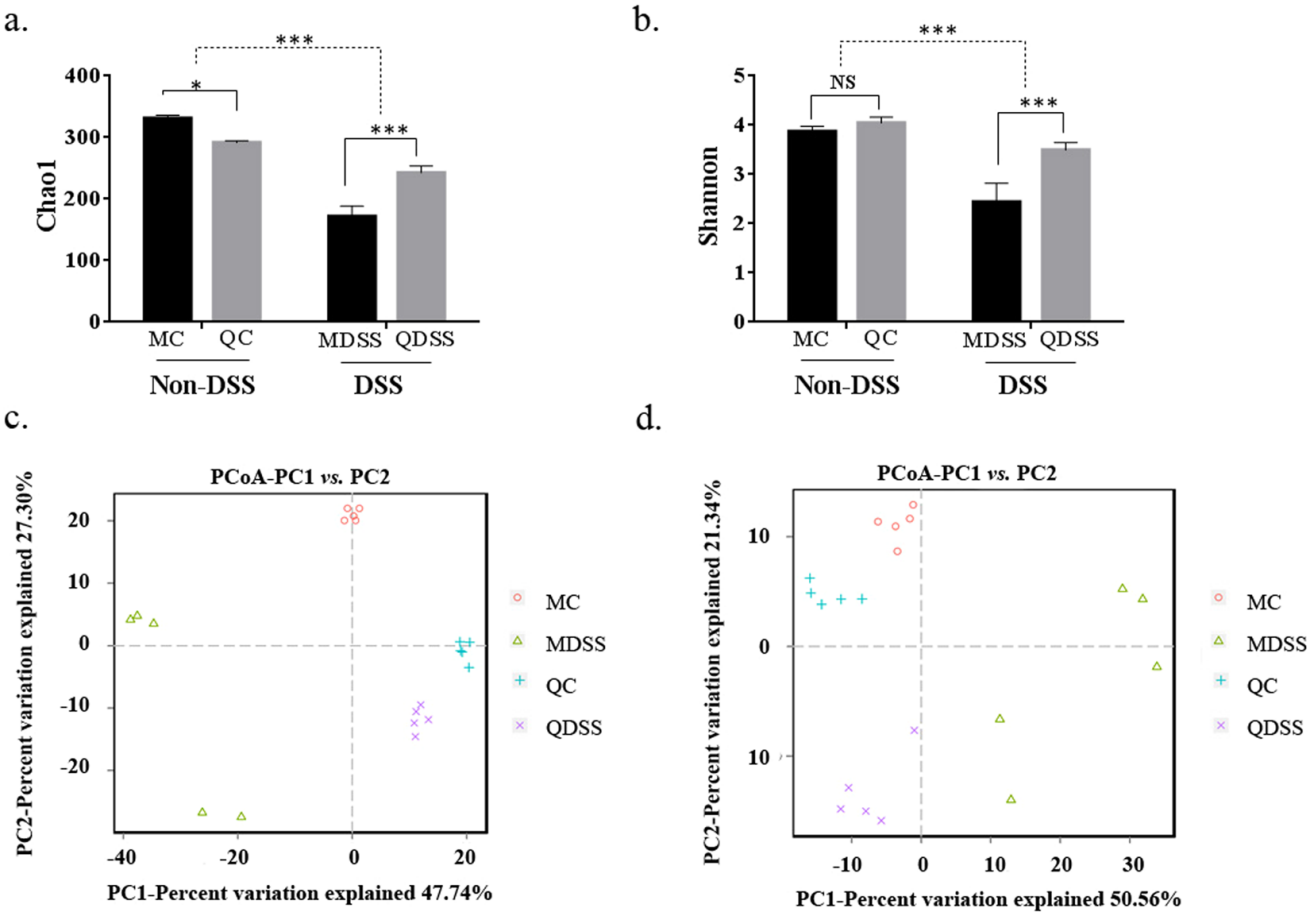
Microbiota diversity indices of the different groups: (**a**) the bacterial richness of microbiota communities estimated by the Chao1 value. (**b**) The bacterial diversity of the microbiota communities estimated by Shannon index; (**c**) PCoA plot of unweighted UniFrac distance values and (**d**) weighted UniFrac distance values. All groups of α diversity indices (Chao1 and Shannon) were analyzed by two-way analysis of variance (ANOVA) followed Fisher’s LSD post hoc tests. *Indicates significant difference with *P* < 0.05, **Indicates *P* < 0.01 and ***Indicates *P* < 0.001; NS indicates lack of statistically significant difference.

As shown in Fig. 2a,b, significant reduction of both microbial species richness (Chao1) and diversity (Shannon) were observed in DSS-treated colitis mice, when compared to non-DSS control groups (Chao1: *P* < 0.001; Shannon: *P* < 0.001). When the comparison was performed for the non-DSS control groups, the Chao1 index was lower in the QC group (*P* = 0.0163), and no difference for the diversity was indicated by the Shannon index (*P* = 0.244). However, after the DSS exposure, both of two indices were significantly higher in mice fed with quinoa when compared to the mice fed the AIN-93M control diet (QDSS vs. MDSS, Chao 1: *P* < 0.001; Shannon: *P* < 0.001).

The community structure of the microbiota was also analyzed using principal coordinates analysis (PCoA) with weighted and unweighted UniFrac distance matrix. As shown in Fig. 2c (unweighted) and Fig. 2d (weighted), both weighted and unweighted UniFrac metrics showed distinct separation of the four treatment groups, which means that DSS exposure and diet are both key influence factors on the composition of gut microbiota.

### Microbial taxonomic structure analysis

**Figure 3:**
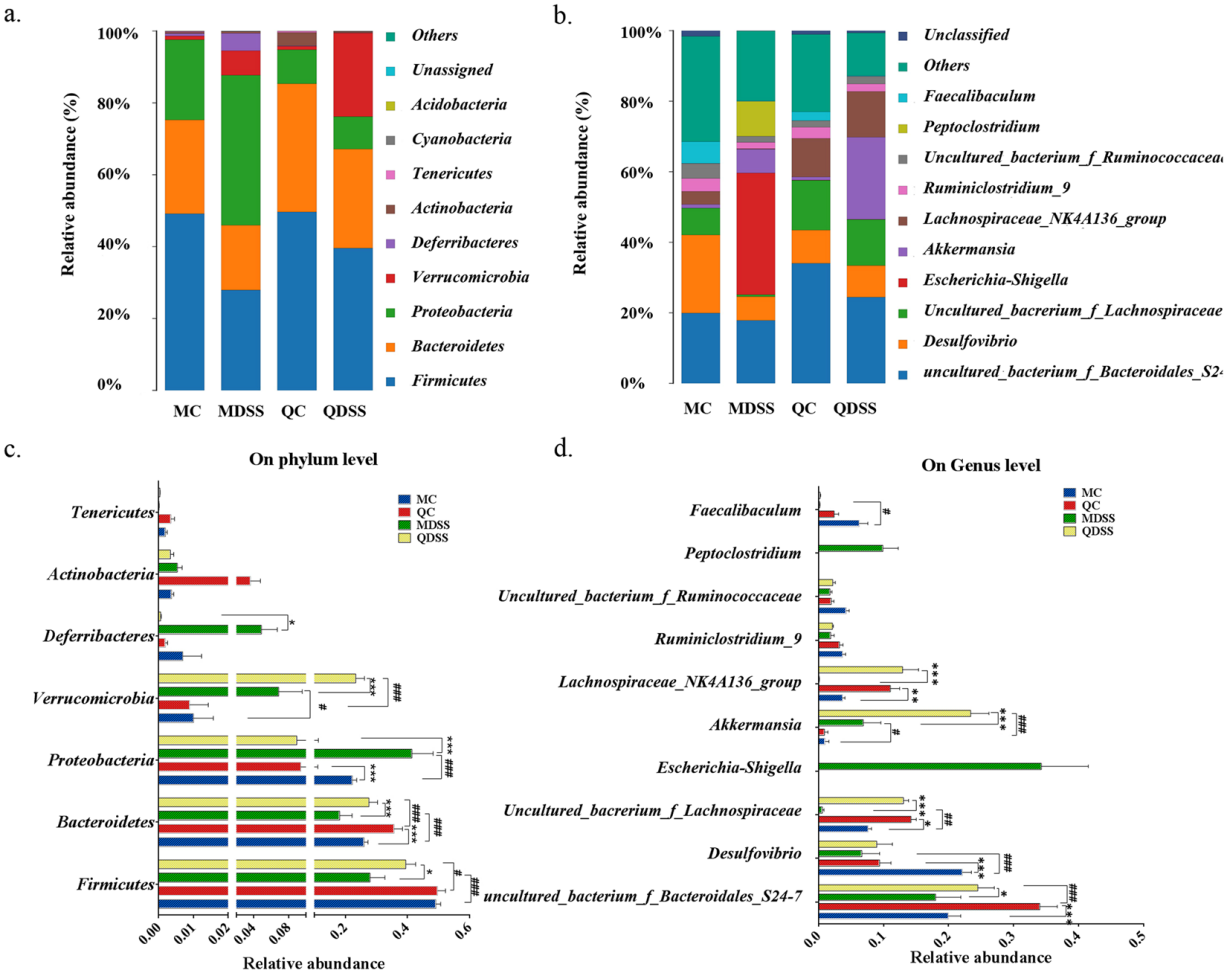
Compositions of microbiota in mice cecum on (**a**) phylum level and (**b**) genus level in all experimental groups and comparisons of relative abundance (percentage of sequences) of the (**c**) main bacterial phyla and (**d**) main bacterial genera. Calculation was based on average relative abundance of main bacterial communities. In (**c**) and (**d**), asterisk indicates difference within same treatment (MC *vs*. QC, MDSS *vs*. QDSS), ^*^Indicates significant difference with *P* < 0.05, ^**^Indicates *P* < 0.01 and ^***^Indicates *P* < 0.001; ^#^Represents a significant difference between same diet with different treatment (MC *vs*. MDSS, QC *vs*. QDSS), ^#^Indicates significant difference with *P* < 0.05, ^##^Indicates *P* < 0.01 and ^###^Indicates *P* < 0.001. NS indicates lack of statistically significant difference.

The microbial taxonomic composition and the details of bacterial taxonomy comparison at the phylum level were demonstrated in Fig. 3a,c, respectively. Phylogenetic classification of OTUs revealed *Firmicutes* and *Bacteroidetes* are the predominant phyla in non-DSS control groups (MC and QC groups). The relative abundances of *Firmicutes* in MC and QC mice were 49.2% and 49.7%, and the abundances of *Bacteroidetes* in MC and QC was 26.1% and 35.8%, respectively. After treatment with DSS, the relative abundances of *Firmicutes* and *Bacteroidetes* were significantly decreased when compared to their non-DSS counterparts. The relative abundance of *Firmicutes* decreased to 28.0% (MDSS) and 39.5% (QDSS), and that of *Bacteroidetes* decreased to 18.2% (MDSS) and 27.6% (QDSS). The magnitude of decrease was much smaller in the quinoa diet groups (QC vs. QDSS), when compared to the AIN-93M control diet groups (MC vs. MDSS), and the relative abundances of these two phyla in the QDSS mice were significantly higher than those in the MDSS mice (*Firmicutes*: *P* < 0.001; *Bacteroidetes*: *P* < 0.001). The phylum *Proteobacteria* expanded substantially and occupied dominantly in the MDSS group (41.4%), significantly higher (*P* < 0.001) than its non-DSS control group (MC, 22.3%), whereas similar proportions of *Proteobacteria* were noted in QC (9.43%) and QDSS (8.96%) groups. It is specifically noteworthy that the proportions of *Proteobacteria* in the QC and QDSS groups were far lower than the proportions in the MC and MDSS diets group. The phylum *Verrucomicrobia* is significantly increased in DSS-treated groups, when compared to its non-DSS counterpart control groups; the magnitude is particularly significant for the quinoa groups (*P* < 0.001). The expansion of *Verrucomicrobia* accompanied with DSS feeding has been detected previously^17^ and might due to certain bacteria in *Verrucomicrobia* can de-polymerize DSS and thus are able to grow in a DSS-rich environment^17,18^.

**Figure 4:**
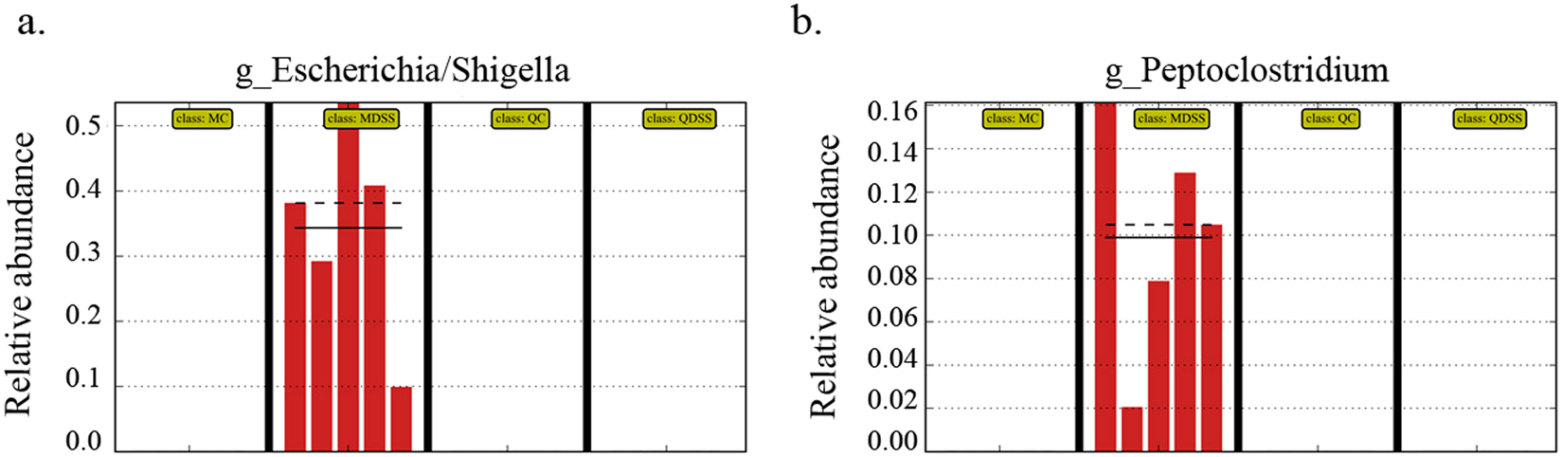
Relative abundance of genus *Escherichia/Shigell*a and *Peptoclostridium* in the sequenced samples (n = 5/group).

We also compared the microbial taxonomic compositions at the genus level, with the results shown in Fig. 3b,d. We first noticed that an uncultured bacterium from family *Bacteroidales S24-7* was significantly higher in quinoa diet groups, regardless of DSS treatment (MC vs. QC, *P* < 0.001; MDSS vs. QDSS, *P* = 0.0145). Several other genera, including the *Lachnospiraceae NK4A136* group, an uncultured bacterium from family *Lachnospiraceae*, *Escherichia/Shigella* and *Peptoclostridium*, were significant changed with DSS treatment in the AIN-93M diet, but the consumption of quinoa compromised their changes. The two genera belonging to family *Lachnospiraceae*, *Lachnospiraceae_NK4A136_group* and the *uncultured_bacterium_f_Lachnospiraceae*, significantly decreased in the MDSS diet when compared to the MC diet (*P* < 0.05), whereas similar high levels were maintained between the QC and QDSS groups. The other two special genera, *Escherichia/Shigella* and *Peptoclostridium*, were only detected in the MDSS group and not the QDSS group (Fig. 4a,b). In particular, *Escherichia/Shigella* increased dramatically and occupied the dominant position in gut microbiota of MDSS mice. The results suggested that intake of the quinoa diet reversed the changes of gut microbiota induced by DSS treatment, including inhibiting the overgrowth of *Escherichia/Shigella* and *Peptoclostridium*, and prompting the growth of two genera from family *Lachnospiraceae*.

In addition, at the genus level we noticed a significant increase of *Desulfovibrio* and a decrease of *Akkermansia* induced by DSS treatment, which to our knowledge, has not yet been reported in any other studies. This might be a characteristic related to DSS exposure, as the similar trend was observed in both AIN-93M and quinoa-based dietary groups.

### LEfSe analysis of cecal microbiota

**Figure 5:**
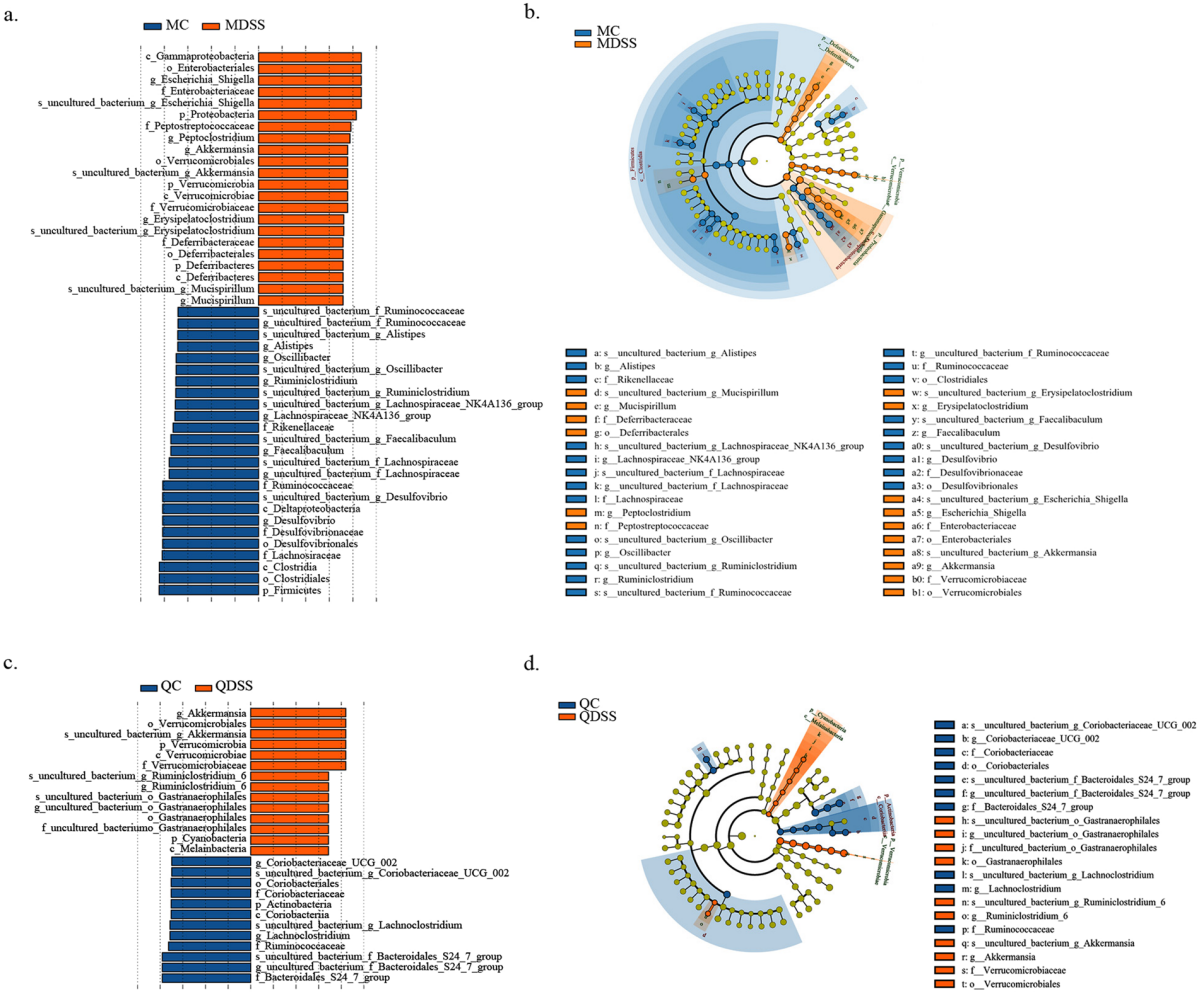
LEfSE was used to identify the representative taxa in different groups. Output showing effect size of significantly enriched taxa in each group when perform comparison between (**a**) MC and MDSS; (**c**) QC and QDSS. Taxonomic cladogram obtained using LEfSe analysis of the 16S sequences from (**b**) MC *vs*. MDSS and (**d**) QC *vs*. QDSS. The differential abundant taxa are presented with designated colors using LEfSe method, Schemes follow the same formatting. The taxa from are colored in blue and orange, respectively. The taxa with nonsignificant changes between the non-DSS and DSS treated mice are colored in yellow. Each small circle’s diameter represents the taxon abundance.

The cecal microbiota in the groups was further analyzed by LFfSe (LDA Effect Size). The LEfSe Cladogram and the histogram of LDA scores are shown in Fig. 5. A total of 47 different taxa (from phylum to species level) were found between the MC and MDSS group, including 25 dominant communities in the MC group and 22 dominant communities in the MDSS group (Fig. 5a,b). The cecal microbiota of mice on the quinoa diet were much more stable after DSS treatment, and only 26 different taxa were found between the QC and QDSS groups, with 12 of them enriched in the QC group and 14 enriched in the QDSS group (Fig. 5c,d).

### Multiplex cytokine profiling of mice plasma

**Figure 6:**
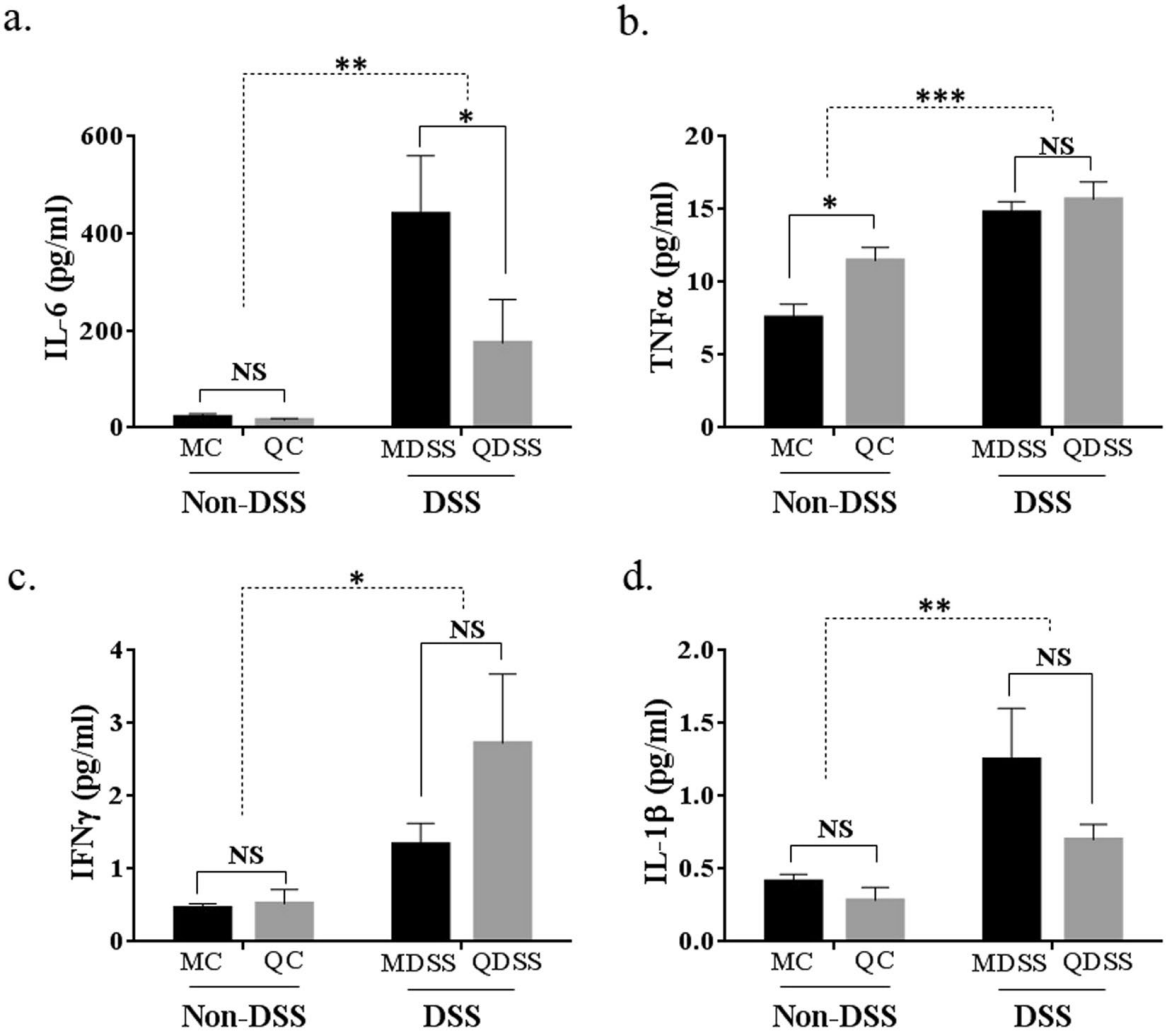
The levels of inflammatory cytokines in the plasma of mice. All data were analyzed by two-way analysis of variance (ANOVA) followed Fisher’s LSD post hoc tests. ^*^Indicates significant difference with *P* < 0.05, ^**^Indicates *P* < 0.01 and ^***^Indicates *P* < 0.001; NS indicates lack of statistically significant difference.

As shown in Fig. 6, the DSS exposure increased circulating levels of all tested inflammatory cytokine levels (MC + QC vs. MDSS + QDSS, *P* < 0.05). When comparing the two diet groups with DSS treatment, plasma level of IL-6 in QDSS was significantly reduced to 174.8 pg/ml, which was much lower than that of the MDSS group (440.0 pg/ml, *P* = 0.028). A decrease was observed for IL-1β (*P* = 0.55) and a surprising increase of IFN-γ (*P* = 0.076) was observed in the QDSS group when compared to the MDSS group, but neither of them reached a statistically significant degree.

## Discussion

Dietary strategies, in opposition to pharmaceutical drugs that generally have side effects, present an attractive means for the prevention of chronic inflammation-related disorders, including IBD^19,20^. Quinoa, a well-known healthy pseudocereal, has a high content of dietary fiber, contains poly-unsaturated fatty acids, and is considered a high-quality source of protein. Furthermore, it contains an abundance of anti-inflammatory phytochemicals^21–23^ and therefore has potential protective effects against inflammation. In the present study, we evaluated the effects of quinoa on IBD using the murine colitis model induced by DSS. The results show that the consumption of quinoa significantly attenuated the clinical symptoms in the DSS-induced colitis model when compared to the condition in the mice fed the AIN-93M control diet, as indicated by reduced body weight loss, better disease activity index, less histological colon damage, and improved inflammatory status (lower plasma IL-6 level).

In particular, the present study aimed to examine the effects of quinoa on the symptoms of colitis and gut microbiota dysbiosis induced by DSS. As quinoa contains several prebiotic components, including polysaccharides and phenolic compounds^16,23^, it may possess the capability to modify gut microbiota. In our present study, PCoA analysis of microbial taxa from cecal content samples distinctly separated the four experimental groups, suggesting a regulatory property of quinoa on gut microbial composition. As expected, DSS treatment significantly reduced species richness and diversity indices (Chao1 and Shannon) of gut microbiota^24^, but the richness and diversity, as indicated by the Chao1 and Shannon indices, were significantly higher in the QDSS group than the MDSS group. Considering controls without DSS treatment, the Chao1 and Shannon indices in the QC group were not higher than those in the MC group; current results suggested that consumption of quinoa compromised the reduction of species richness and diversity induced by DSS treatment, which indicates the positive effect of quinoa on the maintenance of intestinal ecosystems.

Our results indicate a dramatic expansion of the *Proteobacteria* phylum induced by DSS treatment mice fed the AIN-93M diet, whereas there was no relative expansion of *Proteobacteria* by DSS administration in mice fed the quinoa-based diet. The unusual expansion of *Proteobacteria* is often considered as a “microbial signature” of dysbiosis in gut microbiota^25^. Therefore, the inhibitory effect on phylum *Proteobacteria* expansion by quinoa could be potentially beneficial to intestinal health.

It was also noteworthy that DSS treatment induced the bloom of the genera *Escherichia/S*higella and *Peptoclostridium* in mice fed the AIN-93M diet, whereas neither of them were observed in mice fed quinoa. Both *Escherichia/Shigella* and *Peptoclostridium* are generally believed to be pro-inflammatory, as they contains *Escherichia coli* and *Peptoclostridium difficile*, the main pathogens associated with infectious diarrhea^26^. A dramatic decrease was observed for two genera belonging to family *Lachnospiraceae* by DSS treatment in mice fed the AIN-93M diet, whereas the change was not observed in animals fed quinoa (QC vs. QDSS). As the inoculation of *Lachnospiraceae* in germ-free mice can suppress the growth of *Peptoclostridium difficile*^27^, the decrease of *Lachnospiraceae* might be responsible for the expansion of *Peptoclostridium* in the MDSS group.

To our knowledge, this is the first study to analyze the effects of a quinoa-based diet on a colitis animal model. Our data demonstrated that consumption of quinoa modified the dysbiosis of intestinal microbiota, and compromised the clinical symptoms of DSS-induced colitis. The promising results from our animal study warrant further clinical studies to establish the consumption of quinoa as a dietary strategy to improve intestinal health.

## Materials and Methods

### Preparation of Quinoa flour

The quinoa seeds (white quinoa) used in this study supplied by Shandong Center of Crop Germplasm Resources (Jinan, Shandong, China), which planted in Xinzhou, Shanxi, China. Quinoa seeds were cleaned and washed several times until no foam was visible in the water; this was done in order to remove saponins from the outer skin of the seeds. The washed quinoa seeds were dried at 42 °C for at least 12 h, and then ground into quinoa flour. The resulting quinoa flour contained 15.0 ± 0.46% protein, 11.3 ± 0.35% moisture, 4.7 ± 0.41% fat, 61.8 ± 1.15% carbohydrates (in which, 55.6% are starch) and 2.0 ± 0.10% ash.

### Animals and Diets

Forty male C57BL/6 mice (6-wk old) were purchased from Model Animal Research Center of Nanjing University (Nanjing, China), and housed in a facility with a controlled environment (22 ± 2 °C, 55 ± 10% humidity, inverted 12-h daylight cycle) with *ad libitum* access to food and water. Mice were acclimated for one week on a standard chow diet, then randomly assigned into two experimental diets (n = 20 per diet) where they were fed either the AIN-93M purified rodent diet or a quinoa-based diet. In the quinoa-based diet, carbohydrates (cornstarch, maltodextrin, sucrose and cellulose) were replaced with quinoa powder. Since the quinoa powder contains 15% protein and, therefore, 907 g/kg of quinoa contributes to a total of 140 g/kg protein (136 g protein from quinoa and 4 g casein), was added to match the content (140 g/kg of protein casein) in the AIN-93M diet. Detailed compositions are listed in Table S1. After one week of experimental diets, half of the mice in each of the two dietary groups were exposed to DSS (molecular weight 36–50 kDa; MP Biomedicals) in their drinking water (2.5%) for 5 consecutive days, which was then changed to standard tap water from day 6 to day 10 of the study. The DSS solution was freshly prepared every day. The remaining half of the mice, non-DSS controls, received standard tap water through the whole course of the study. Thus, the study consisted of 4 groups (10 animals/group): (1) MC, AIN-93M diet without DSS treatment; (2) QC, quinoa diet without DSS treatment; (3) MDSS, AIN-93M diet with DSS treatment; (4) QDSS, quinoa diet with DSS treatment. The animal protocol was approved by Animal Care and Use Ethics Committee of Shandong Normal University (Permission Number: AEECSDNU2018004), and all of the procedures were conducted in accordance with the rules for animal welfare of Shandong Normal University.

### Evaluation of disease activity index (DAI)

Body weight, stool consistency and rectal bleeding were recorded every two days. The DAI was calculated by combining the measured scores of body weight, stool consistency and stool bleeding according to previously described methods^28^. The details of each scores are listed in Supplementary Materials (Table S2).

### Tissue Collection

On day 10, the mice were anesthetized with isoflurane inhalation, and blood was collected with a capillary tube containing 1% heparin sodium solution by orbital puncture and then centrifuged at 6000 rpm for 10 minutes to obtain plasma. After termination by cervical dislocation, the abdomen was opened, the large intestine was removed and placed on an ice plate, and then cecal content was collected, snap-frozen in liquid nitrogen, and stored at −80 °C until further analysis. The colon length (measured from the colo-cecal junction to the anus), spleen weight and liver weight were recorded, and the colon tissue was collected for histopathological analysis.

### Histopathological Analysis

After fixation for 48 h in 10% formalin, the colon tissues were processed for paraffin embedding, with 5 μm colon sections stained with hematoxylin and eosin (H&E). Eight randomly selected fields were viewed under a light microscope, and each view given scores according to previous descriptions^19,29^. In brief, scores were given based on inflammation severity (0–3), inflammation extent (0–3), and crypt damage (0–4), with the total histologic scores then calculated by adding the scores of all three parameters, resulting in a maximum potential score of 10. The detailed description of score standards are listed in Supplementary Materials (Table S3).

### Analysis of cecal microbiota composition by 16S ribosomal RNA gene sequencing

Total bacterial DNA was isolated from frozen cecal contents using a QIAamp DNA Stool Mini Kit (Qiagen, Valencia, CA). The region V3–V4 of the 16S rRNA gene was amplified and DNA libraries were constructed according to those previously described^30^. The paired-end sequencing with a read length of 2 × 250 bp was performed on the Illumina MiSeq platform (Illumina, Inc, San Diego, California). After the demultiplexed paired-end reads were joined (FLASH v1.2.7)^31^, quality filtered^32^, and chimerism removed^33^, the resulting tags were assigned to OTUs using UCLUST (version 1.2.22)^34^ with a 97% threshold of pairwise identity. The identified taxonomy was then aligned against the Silva reference database (Release128, http://www.arb-silva.de)^35^. The α-diversity analysis was performed using Mothur (version v.1.30, http://www.mothur.org/)^36^, the Chao1 index was used to characterize species richness and the Shannon diversity index was used to characterize species diversity. Two-dimensional principal coordinate analyses (PCoA) plots, including weighted and unweighted Unifrac, were used to assess the variation (β-diversity distance) between experimental groups. The raw Illumina read data for all samples were uploaded into SRA at NCBI under accession number SRP141297.

### Multiplex cytokine profiling in mice plasma

The plasma samples were centrifuged at 4 °C at 3000 rpm for 10 minutes, the supernatant was collected and diluted 2-fold for further analysis. Concentrations of IL-1β, TNF-α, IL-6 and IFNγ were determined using the Proinflammatory Panel 1 (mouse) V-PLEXTM Kit and QuickPlex SQ 120 (Mesoscale Discovery, Rockville, MD) according to manufacturer’s protocols. Cytokines were expressed as ng/ml plasma.

### Statistical analysis

Comparisons of various anatomical measurements among the four groups were made by two-way analysis of variance (ANOVA), followed by Fisher’s LSD *post hoc* tests. The results were considered statistically significant when *P* < 0.05. The results are presented as the mean ± SEM. GraphPad Prism version 6.00 for Windows (GraphPad Software, Inc., La Jolla, California) was used for all calculations. As previously described^37^, the linear discriminant analysis (LDA) effect size (LEfSE, https://huttenhower.sph.harvard.edu/galaxy/) was applied to identify bacterial communities responsible for the differences of cecal microbiota compositions between different groups, using an LDA score threshold of >4.0.

## Electronic supplementary material

Supplementary Materials
