# Supplementary material for: Quinoa whole grain diet compromises the changes of gut microbiota and colonic colitis induced by dextran Sulfate sodium in C57BL/6 mice
Source: Sci Rep. 2018 Oct 8;8:14916. doi: 10.1038/s41598-018-33092-9 (PMC6175902; doi:10.1038/s41598-018-33092-9)
Supplement: Supplementary file 1 — Supplementary Materials [file 41598_2018_33092_MOESM1_ESM.docx]

Title: Quinoa whole grain diet compromises the changes of gut microbiota and colonic colitis induced by dextran Sulfate sodium in C57BL/6 mice

Wei Liu^1, 2, 3^, Yu Zhang1, Bin Qiu^2^, Shoujin Fan^4^, Hanfeng Ding ^1, 4*^ & Zhenhua Liu^3*^

1 Shandong Center of Crop Germplasm Resources, Shandong Academy of Agricultural Sciences, Jinan 250100, China

2 Institute of Agro-Food Science and Technology, Shandong Academy of Agricultural Sciences, Jinan 250100, China

3 School of Public Health and Health Sciences, University of Massachusetts, Amherst 01003, US

4 College of Life Science, Shandong Normal University, Jinan 250014, China

***Corresponding Author:**

Hanfeng Ding: [dinghf2005@163.com](mailto:dinghf2005@163.com); Tel: +86-531-66659996, Fax +86-531-66659995

Zhenhua Liu: zliu@nutrition.umass.edu; Tel: 1-413-545-1075; Fax: 1-413-545-1074

**Keywords:** Quinoa; Inflammatory bowel disease; Gut microbiota; 16S rRNA gene sequencing

**Table S1:** Composition of AIN-93M and Quinoa-based diets used in this study.

| **Composition** | **Diet (g/1000 g)** | |
| --- | --- | --- |
|  | **AIN-93M** | **Quinoa-based** |
| Casein | 140 | 4 |
| L-Cystine | 1.8 | 1.8 |
| Cornstarch | 495.69 | 0 |
| Maltodextrin | 125 | 0 |
| Sucrose | 100 | 0 |
| Cellulose | 50 | 0 |
| Soybean Oil | 40 | 40 |
| Mineral mixture(AIN-93M-MX)* | 35 | 35 |
| Vitamin mixture(AIN-93-VX)* | 10 | 10 |
| Bitartrate Choline | 2.5 | 2.5 |
| Tert-butylhydroquinone | 0.008 | 0.008 |
| Quinoa powder | 0 | 906.96 |
| Total calories(kcal) | 3849.96 | 3592.85 |

***** The compositions of mineral and vitamin mixture were followed the description of Reeves et al., 1993.

**Table S2.** Disease Activity Index Assessment Standards

| **Score** | **Body Weight** | **Stool characteristic** | **Rectal bleeding** |
| --- | --- | --- | --- |
| 0 | No Body weight loss or body weight loss less than 1% | Normal stool consistency | negative hemoccult |
| 1 | Body weight loss between 1% and 5% | Soft stools | positive hemoccult |
| 2 | Body weight loss between 5% and 10% | Very soft stools | traces of blood |
| 3 | Body weight loss more than 10% | Watery stools | Visible rectal bleeding |

**Table S3.** Histological Scores of Colon Damage

| **Score** | **Inflammation severity** | **Inflammation extent** | **Crypt damage** |
| --- | --- | --- | --- |
| 0 | None | None | None |
| 1 | Mild | Mucosa | Basal 1/3 damaged |
| 2 | Moderate | Mucosa and submucosa | Basal 2/3 damaged |
| 3 | Severe | Transmural | Crypts lost, surface epithelium present |
| 4 | - | - | Crypts and surface epithelium lost |
